# The composition of heavy minerals of the sandy lands, Northeast China and their implications for tracing detrital sources

**DOI:** 10.1371/journal.pone.0276494

**Published:** 2022-10-20

**Authors:** Lei Sun, Yuanyun Xie, Chunguo Kang, Yunping Chi, Peng Wu, Zhenyu Wei, Siqi Li, Qian Zhao, Shuo Liu

**Affiliations:** 1 College of Geographic Science, Harbin Normal University, Harbin, China; 2 Heilongjiang Province Key Laboratory of Geographical Environment Monitoring and Spatial Information Service in Cold Regions, Harbin Normal University, Harbin, China; 3 College of Geography and Tourism, Harbin University, Harbin, China; Institute of Earth and Environment, Chinese Academy of Sciences, CHINA

## Abstract

Comprehending heavy mineral composition of the sandy land in Northeast China (NESL) is of great significance for interpreting generation, pathways, source and geochemistry of sediments in this area. To this end, the fine-grained (<63 *μm*) aeolian-fluvial sediments and loess deposits, which were taken from the Onqin Daga Sandy Land, the Horqin Sandy Land, the Hulun Buir Sandy Land and the Songnen Sandy Land, and from the downwind loess section (L_1_), respectively, were analyzed to construct the heavy mineral data set of NESL source and sink and to evaluate feasibility of the heavy mineral method in tracing the source of aeolian dust in Northeast China. Additionally, the <63 μm, 63–125 μm and 125–250 μm fractions of the fluvial sands from the different Balan River reaches having a same source, were analyzed to valuate the impact of the river transport-sedimentation process on the heavy mineral composition. The results show that the NESL shows moderate similarities in the heavy mineral composition, with ilmenite, epidote, zircon and amphibole as the primary minerals. In the source-to-sink system in the NESL, limited by sedimentary differentiation, post-deposition alteration and similar source material composition, the heavy mineral composition of the loess and that of sandy-land sources does not well correlate, indicating single heavy mineral method is incapable of unequivocally detecting loess sources when not considering the physical geographical conditions. The sediments in the different Balan River reaches show clear diversities and grain-size dependency in heavy minerals composition, indicating the river transport-deposition processes exert a clear control on the heavy-mineral composition in the sediment downstream. Both a wide grain-size window and more numbers of samples are needed to obtain a complete heavy-mineral picture in the source area.

## Introduction

The arid and semi-arid deserts and sandy lands of northern China are important potential sources of Asian aeolian dust [1–6]. Fine-particle dust deflated from the potential sources is transported by atmospheric circulation and prevailing winds to accumulate, in the downwind areas, as loess and paleosol deposits over the glacial-interglacial periods, respectively [5, 7]. The loess-paleosoil sequences are widely used to study the phased aridification process and natural environment changes in inland Asia [8–14]. The coupling between sandy land and loess is essential for understanding dust source-to-sink relations.

At present, some extensively-used indicators, e.g., element geochemistry, Sr-Nd isotopes and zircon U-Pb age spectrum, are served for tracing aeolian dust sources [12, 15–19]. Heavy minerals, characterized by high density and strong weather resistance under arid climate conditions, well retain the characteristics of the source-area parent rocks and are provenance-sensitive indicators. Therefore, heavy minerals have unique advantages when analyzing the source of sediments and provide a high-resolution method for identifying the source of sediments [20–25]. Despite this, only few cases are from the application of heavy minerals to aeolian dust source tracing [11, 26–32].

The study of heavy minerals in sandy land areas as potential dust sources, an important basic work for Asian aeolian system research, provides a better understanding of the formation and evolution of sandy lands, as well as of the formation mechanism, source and migration paths of the sand-land materials [19], and has important significance for reconstructing the evolution of past atmospheric circulation. However, there are still some factors affecting applications of heavy minerals to detecting aeolian dust sources, e.g., mechanical abrasion during transport, dynamic sorting, post-deposition alteration and amount of samples [24, 30, 33–37].

**Fig 1:**
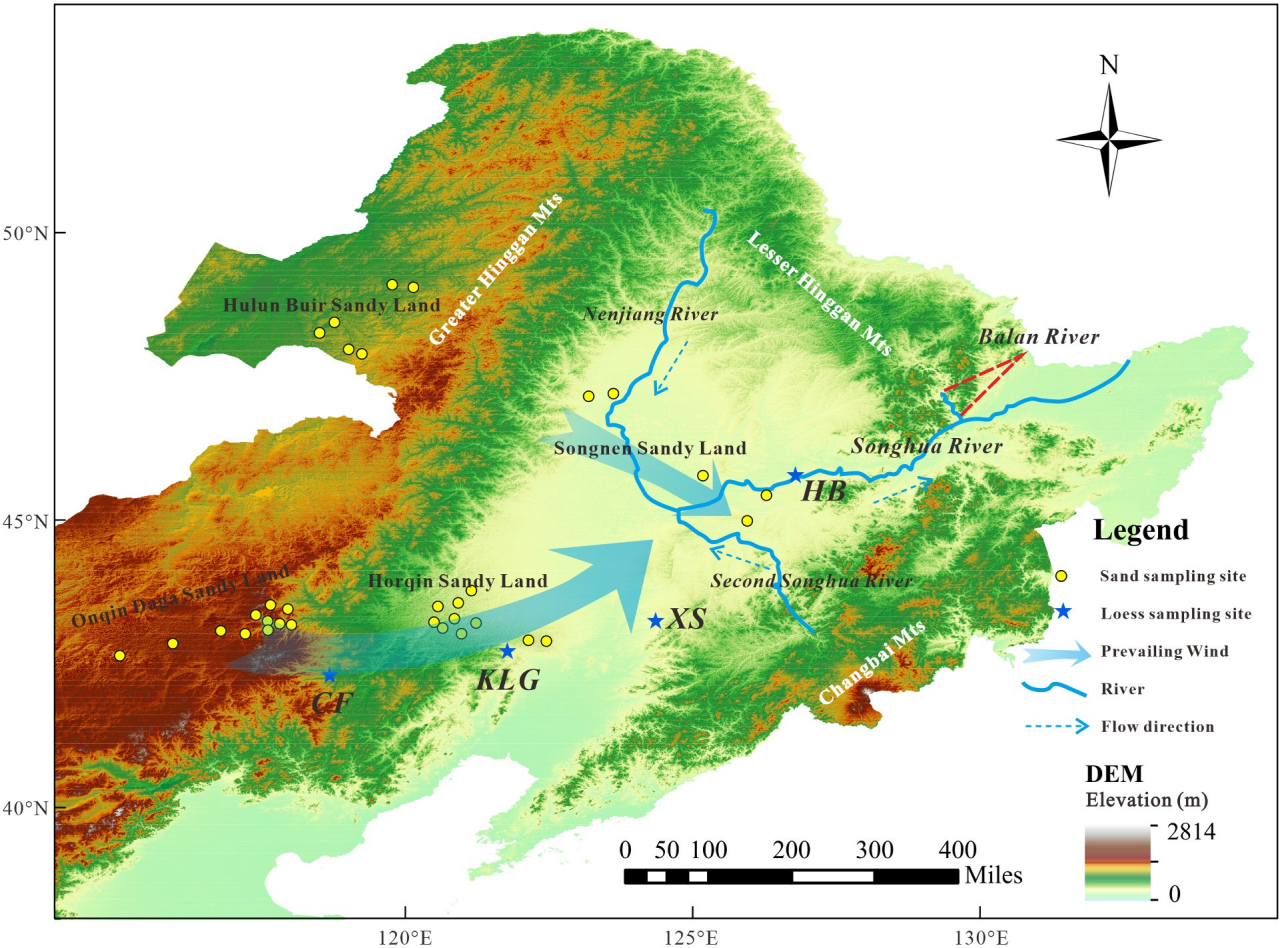
DEM map of the Northeast China plain. The yellow-filled circles are the sampling sites in the dune fields in this study, and the blue stars are sampling sites for the loess profiles. Abbreviations for the loess profiles: CF = Chifeng; KLG = Kulungou; XS = Xingshan; HB = Harbin. The original basemap was obtained from Natural Earth (http://www.naturalearthdata.com/) and was further processed using software ArcGIS 10.4 version. Digital elevation model was from the U.S. Geological Survey (https://www.usgs.gov/).

A detailed study for the heavy-mineral composition in the sandy lands in Northeast China (NESL) (Fig 1) which is located at an important monsoon fringe area, is of great significance to the recognition of regional dust systems. The characteristics, provenance, evolution, and paleoclimatic evolution in the Onqin Daga Sandy Land (ODSL), the Hulun Buir Sandy Land (HLSL), the Songnen Sandy Land (SNSL) and the Horqin Sandy Land (HQSL) have been performed through analysis of sediment grain size, geochemistry, sporopollen, and animal fossils, etc [38–45]. Additionally, some studies in the region, e.g., the animal and plant fossils [46, 47], regional structural features [48, 49], mineral deposit geology [50–52] and geological relics [53, 54], were also involved. Although some achievements have been made in the heavy mineral-based aeolian dust source tracing in northwest China [11, 26–28], few works have been carried out in terms of heavy-mineral aeolian dust system in northeast China, and whether the heavy-mineral method can be used to effectively trace dust sources in the region remains to be verified.

In this study, the fine-grained (<63 μm) aeolian-fluvial sediments, which were taken from the NESL, i.e., the ODSL, the HQSL, the HLSL and the SNSL, were analyzed to construct heavy mineral datasets of the NESL. Additionally, the loess deposits in northeast China (NE loess) were also collected to evaluate the feasibility of the heavy mineral method in tracing the source of aeolian dust in Northeast China. Also, the Balan River, a branch of the Songhua River, was taken as an example to evaluate, under a condition of the same provenance, the influence of river transport-deposition processes on the heavy mineral composition. This work provides an important reference for a well understanding of the source-to-sink system in the NESL.

## Environmental setting

The deserts in northern China, the west-east-trending largest mid-latitude temperate arid inland regions in the world, are adjacent to the Central Asia arid region to the west and the Mongolia arid region to the north, and divided by the Helan Mts into two parts, with the temperate continental climate to the west and the Asian temperate monsoon climate zone to the east.

The NESL, situated at the easternmost edge of the northern desert, comprises the ODSL, the HQSL, the HLSL and the SNSL [55] (Fig 1). The ODSL, 52000 km^2^ and an average altitude of about 1100 m asl, is located at the southern side of the Xilin Gol grassland in central Inner Mongolia, with mostly fixed or semi-fixed dunes. The HQSL, 62432 km^2^, is the largest sandy land in China, mainly distributed in the flood plain of the West Liaohe River, and mainly comprises semi-fixed dunes accounting for 50%~60%, mobile dunes (25%~35%) and fixed dunes (10%~15%). The HQSL is linked by the West Liaohe River to the ODSL, and both border the Central Asian Orogenic Belt (CAOB) and the North China Craton (NCC). The HLSL, with an area of 7435 km^2^, is one of the highest latitude sandy lands in China and located at the western Great Hinggan Mts, southern edge of the CAOB and Ergun block. It is developed from a fluvial-lacustrine plain, with the fixed and semi-fixed dunes distributing along the eastern areas of the Hulun Lake. The SNSL, 8355.95 km^2^, is located in the central and western Songnen Plain, most of which are located on Songliao block in the CAOB [19, 45]. The wetland area is widely distributed and the water system is developed in the Songnen Plain, and the sandy land is distributed along the flood plain and river terraces of the Nenjiang River and the Songhua River [56].

The NE loess is sparsely distributed at the margin of sandy lands, especially in Tongliao and Chifeng areas (Fig 1). Additionally, loess accumulations can also sparsely outcrop in Chaoyang in Liaoning province and Siping in Jilin province, located at the southeast and northeast of the HQSL, respectively. Loess deposits linked with the SNSL were only discovered in the Harbin area in Heilongjiang province (Fig 1).

The Balan River, a branch of the Songhua River (Fig 1), is located at the transition between the Songnen Plain and the Sanjiang Plain. The river originates from the Lesser Hinggan Mts and joins into the mainstream in Yilan, and has a length of 108 km and a total drainage area of about 2075 km^2^, with few tributaries.

## Materials and methods

Quaternary loose sediments in the NESL are mainly composed of river-aeolian sand, followed by small amounts of lacustrine sediments [55]. River sand and aeolian sand are sufficiently mixed by wind and hydraulic transport and thus their fine-grained fractions are capable of representing the average composition of a comparatively large area [19, 45]. A total of 33 aeolian-fluvial sand samples were collected throughout the NESL, 13 samples from the ODSL, 9 samples from the HQSL, 6 samples from the HLSL, and 5 samples from the SNSL. Details for the samples are described in Supporting Information S1 Table. Fine particle fractions (<63 μm) were extracted from all the samples by dry sieving. The reason for this operation is to evaluate the feasibility of heavy mineral components in the sandy lands in tracing aeolian dust sources, because only the <63 μm grain-size fractions can be transported in suspension in the air [19, 57–60] (and the <63 μm silt-clay fractions are dominant in loess deposits. Such an operation, therefore, allows inter-sample heavy mineral comparison in the same grain-size ranges.

To evaluate the feasibility of heavy mineral composition in tracing the source of the aeolian dust in Northeast China, 13 loess samples were collected (see Fig 1 for sampling location), all of which are the last glaciation (L_1_) loess, namely Chifeng loess (4 samples), Kulungou loess (4 samples), Harbin loess (4 samples) and Xingshan loess (1 sample). All loess samples were wet-screened to obtain <63 μm fractions for heavy mineral analysis.

The Balan River is sampled downstream for evaluating the influence of river transport-deposition processes on heavy mineral composition under the condition of a single, stable and identical source. The reason for selecting the Balan River is that the river is featured by short course, few tributaries and identical detrital source. A total of 8 fluvial sand samples from point bar were collected from the interval from middle reach to river mouth, into which no tributaries join, with a river length of 42 km and the height difference of 80 m. The collected bulk samples were air-dried and dry-sieved using standard sieves to separate the <63μm, 63–125 μm and 125–250 μm grain-size fractions for heavy mineral analysis.

The sub-samples for heavy mineral analysis were air-dried and then weighed. The heavy parts were extracted by elutriation, and the remaining tail sand and elutriation sewage were weighed to calculate the loss rate. The light and heavy minerals were separated by tribromomethane (density 2.89 g/cm^3^) [31, 61], and the separated samples were washed repeatedly by alcohol, dried at a constant temperature of 60°C and weighed again, and finally, the content of heavy mineral parts was obtained. The separated heavy minerals are identified by randomly selecting 10 fields of view under the solid microscope by strip method, and the average value is taken to reduce the analysis error. Individual heavy mineral grains need to be sliced and polished for further identification under a polarizing microscope. Nearly up to 1000 grains from each sub-sample were counted, and the percentage of heavy minerals was calculated based on the weight.

Data visualization technology is effective in interpreting large sample data sets [62]. To visually reflect the similarity between samples, we standardized the heavy mineral data and showed it through multi-dimensional scaling (MDS) analysis. The Euclidean distance between samples calculated by heavy mineral data reflects the between-sample differences [62]. In a word, the smaller the distance in MDS space, the greater the similarity between the two objects. In this study, the 34 sand and 13 loess samples are represented, and each sample has 20 heavy mineral species as their properties. SPSS software was used to operate the non-metric MDS.

## Result

### Heavy mineral characteristics for the sandy lands in Northeast China

**Fig 2:**
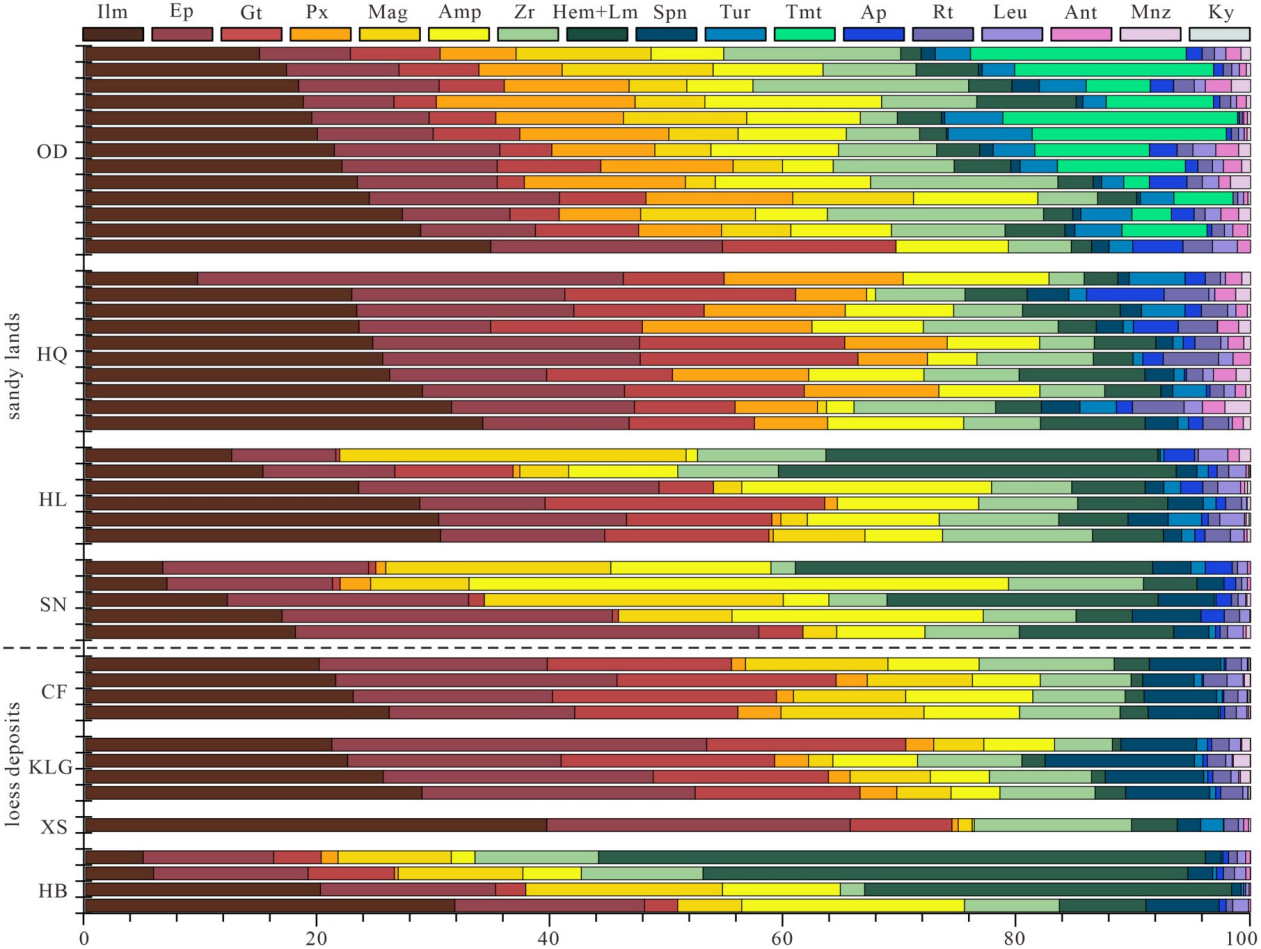
Heavy mineral abundances (wt.%) of the <63 μm fraction in the dune fields and loess deposits in Northeast China. Systematic mineral abbreviation list: Ilm = ilmenite, Ep = epidote, Gt = garnet, Px = pyroxene, Mag = Magnetite, Amp = amphibole, Zr = zircon, Hem = hematite, Lm = limonite, Spn = sphene, Tur = tourmaline, Tmt = titanomagnetite, Ap = apatite, Rt = rutile, Leu = leucoxene, Ant = anatase, Mnz = monazite, Ky = kyanite. Weathered debris is not listed in the figure. Abbreviations for the sandy lands: OD = Onqin Daga Sandy Land; HQ = Horqin Sandy Land; HL = Hulun Buir Sandy Land; SN = Songnen Sandy Land. Abbreviations for the loess profiles are as Fig 1.

The heavy mineral results of the NESL were shown in Fig 2 and S2 Table, and a total of 23 heavy mineral species were identified, including zircon, apatite, kyanite, rutile, anatase, leucoxene, monazite, sphene, garnet, tourmaline, amphibole, epidote, ilmenite, hematite+limonite, pyroxene, magnetite and titanomagnetite. Among them, the authigenic pyrite, moissanite, chromite spinel, chalcopyrite, siderite and gold are not statistically meaningful due to occasional presence in certain one sample, and are not involved in the analysis in this study. In this study, the species with weight percentages exceeding 6% are considered as primary minerals, 1%-6% are secondary minerals, and <1% are trace minerals.

The ODSL is characterized by the primary minerals of ilmenite, epidote, zircon, titanomagnetite, pyroxene, amphibole, magnetite and garnet (Fig 2). The secondary minerals are hematite+limonite, tourmaline, apatite and anatase. less abundant (commonly <1%) but common occurring minerals are rutile, leucoxene, sphene and monazite. The HQSL manifests similar heavy mineral characteristics to the ODSL, with ilmenite, epidote, garnet, pyroxene, amphibole and zircon as the primary minerals. The secondary minerals are hematite+limonite, rutile, tourmaline, apatite, sphene and anatase. Monazite, leucoxene and magnetite are trace minerals (Fig 2).

The primary heavy minerals for the HLSL are ilmenite, hematite+limonite, epidote, garnet, amphibole, zircon, magnetite, with sphene, leucoxene, tourmaline, apatite and rutile as the secondary minerals, and pyroxene, anatase, monazite and kyanite as the trace minerals (Fig 2). The SNSL is featured dominantly by epidote, amphibole, hematite+limonite, magnetite, ilmenite and zircon, with sphene, apatite and garnet as the secondary minerals, and leucoxene, rutile, pyroxene, tourmaline, anatase and monazite as the trace minerals (Fig 2).

### Heavy mineral composition of the loess in Northeast China

The heavy-mineral composition for the NE loess sediments was listed in S3 Table and graphically drawn in Fig 2. The loess sediments share a similar heavy mineral composition to the NESL, collectively with ilmenite, epidote and zircon as the primary mineral types. The other primary heavy minerals in the loess deposits also include garnet, magnetite and amphibole for the Chifeng loess, garnet and sphene for the Kulungou loess, garnet for the Xingshan loess, and hematite+limonite, magnetite and amphibole for the Harbin loess.

### Heavy mineral characteristics in the Balan River

**Fig 3:**
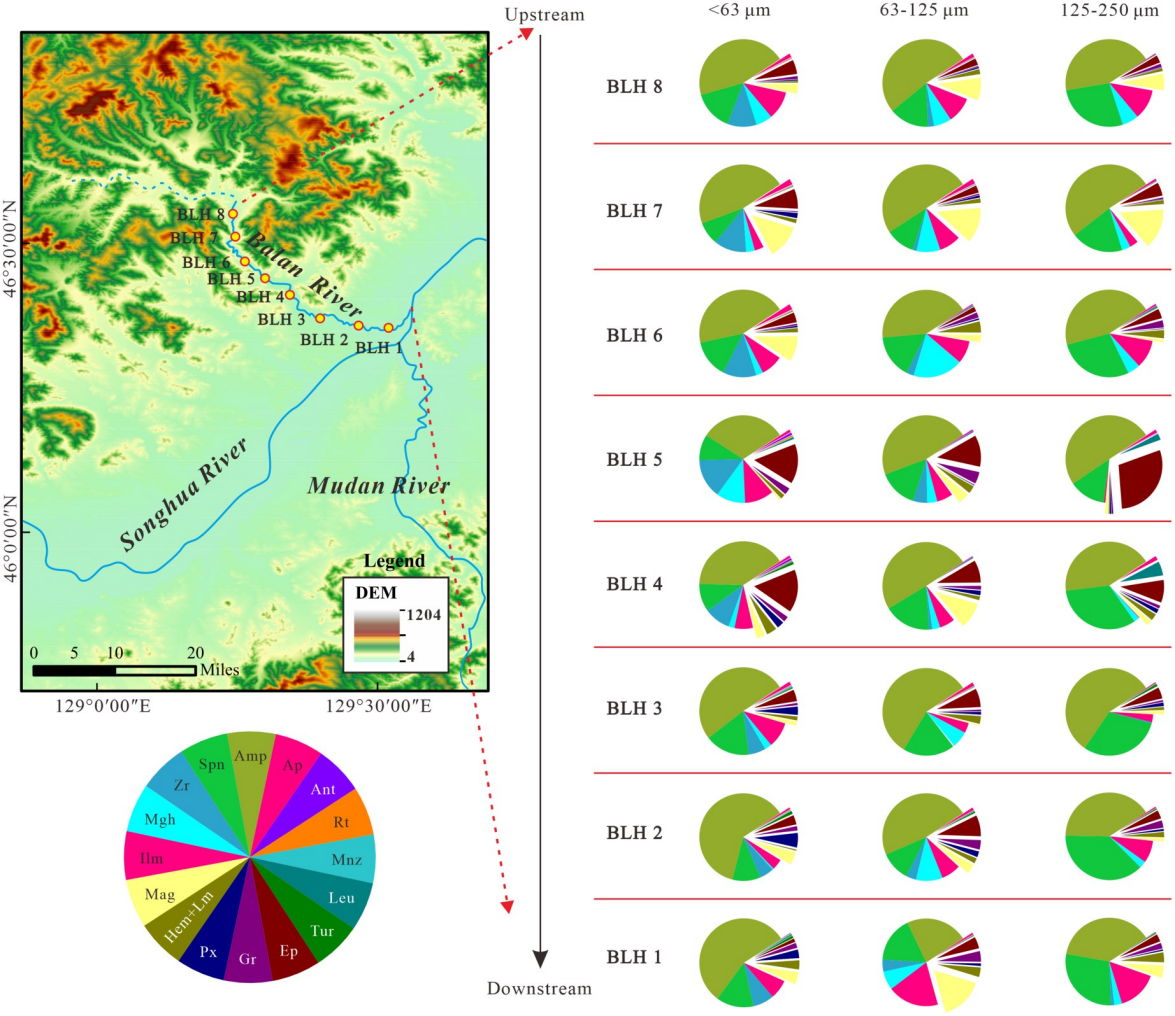
Heavy mineral abundances (wt.%) of three fractions in Balan River basin, Northeast China. Systematic mineral abbreviation list: Amp = amphibole, Spn = sphene, Zr = zircon, Mgh = maghemite, Ilm = ilmenite, Mag = Magnetite, Hem = hematite, Lm = limonite, Px = pyroxene, Gt = garnet, Ep = epidote, Tur = tourmaline, Leu = leucoxene, Mnz = monazite, Rt = rutile, Ant = anatase, Ap = apatite. Weathered debris is not listed in the figure.

The heavy-mineral characteristics for the Balan River were listed in S4 Table and graphically drawn in Fig 3. The three fractions are characterized collectively by the primary heavy minerals, e.g., amphibole, sphene and ilmenite, and the other primary heavy minerals are different in the different grain-size fractions, with zircon and epidote in the <63 μm fraction, maghemite and magnetite in the 63–125 μm fraction, and epidote in the 125–250 μm fraction.

## Discussion

### Evaluating application of heavy minerals to tracing of the aeolian dust sources

**Fig 4:**
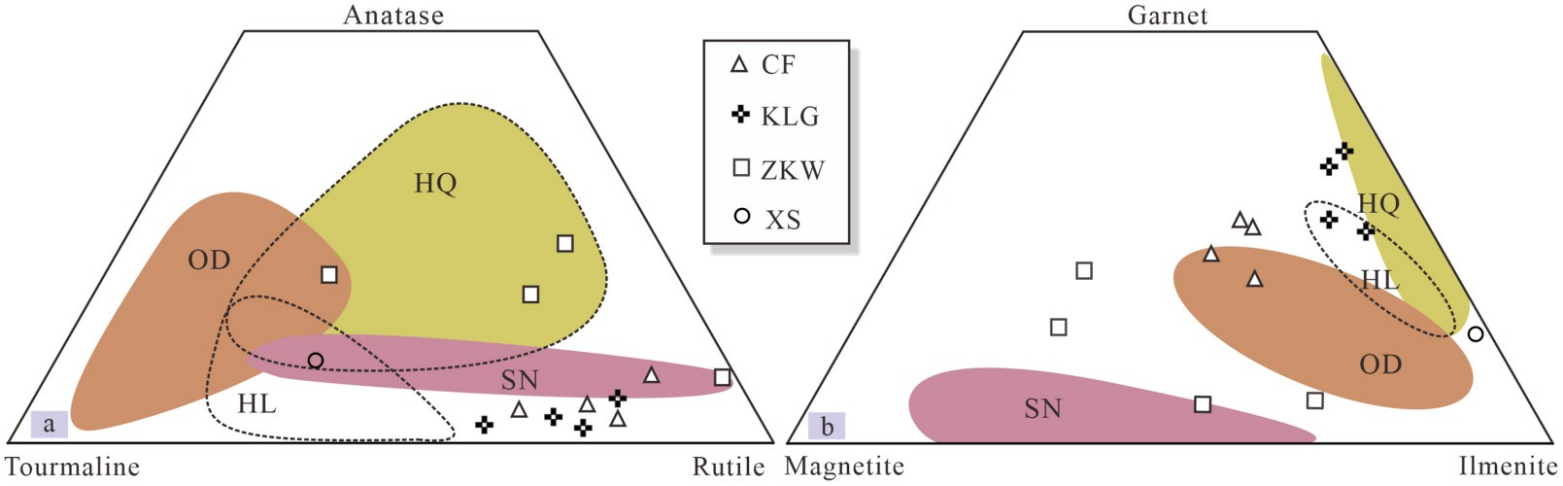
Ternary plot comparing the heavy mineral abundances between the sandy lands and loess deposits, Northeast China. Remarkably, the stable heavy minerals can not give a clear bond between the loess deposits and their respective sources. Details are referred to the text. Abbreviations for the sandy lands are as Fig 2.

The difference in the material composition of potential source areas is a key for tracing aeolian dust sources [19, 45]. The NESL, especially the ODSL and the HQSL, exhibits a certain extent of similarity in heavy mineral species, with ilmenite, epidote, zircon and amphibole as the primary minerals (S2 Table). These sandy lands, however, are slightly different in some characteristic minerals. For example, titanomagnetite is found only in the ODSL, whereas magnetite is almost absent in the HQSL; the HQSL has substantially high contents of garnet, followed by the ODSL and the HLSL; the HLSL and SNSL have a higher content of hematite+limonite, while that of the ODSL and HQSL is lower. Therefore, these characteristic stable heavy minerals (e.g., titanomagnetite, magnetite, garnet, and hematite+limonite) can be used to trace loess provenance in Northeast China, as seen in Fig 4a. Additionally, the sandy lands, to a certain extent, are also distinct in some mineral phase, e.g., pyroxene, tourmaline, rutile and anatase (Fig 4b).

In ternary plot constructed by the above-mentioned characteristic stable heavy minerals as depicting characteristics of the NESL and NE loess (Fig 4a), despite the NE loess being separated into the respective different areas, the NE loess cannot be well linked to their respective dust sources, except the Chifeng loess falling into or next to the ODSL field. This observation indicates that the characteristic stable heavy minerals do not well constrain source areas of the NE loess. Some unstable heavy minerals, like pyroxene and amphibole, are comparatively depleted in the loess samples relative to their dust source areas (S3 Table) due to the post-deposition weathering process. However, some extremely stable heavy minerals, such as tourmaline, rutile and anatase, occur in small or trace amounts in the dust source areas and in much lower abundances in the loess samples. For example, tourmaline is found in trace amounts or deficient in some loess samples. In Fig 4b, except three samples from the Harbin loess and one sample from the Xingshan loess, all sample data are projected tightly together, suggesting weak application of the extremely stable heavy minerals to detection of aeolian dust sources in northeast China.

**Fig 5:**
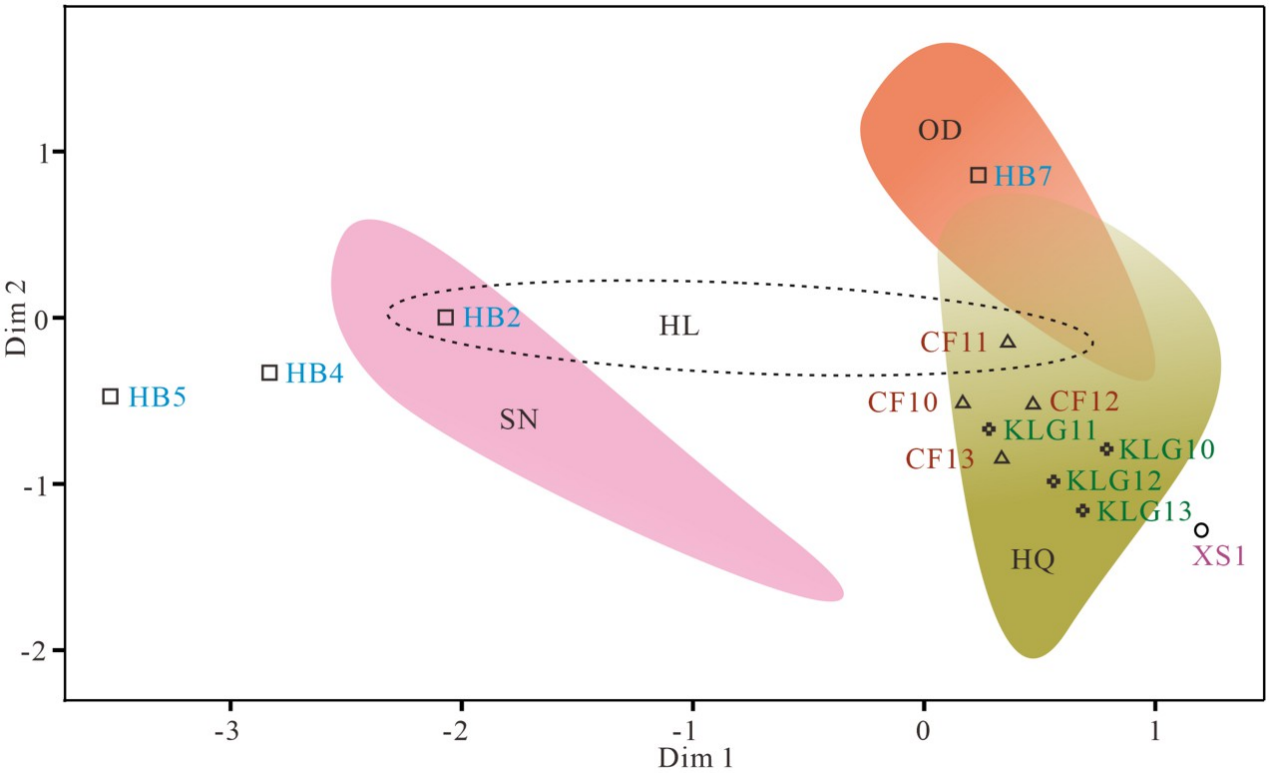
Multi-dimensional scaling (MDS) plot showing the relationship between the studied samples based on heavy mineral abundances. The defined areas represent the distribution scope of the each sandy land. The labelled points represent the loess sites. Note that MDS plot can not well associate the loess deposits with their respective sources. See text for details. Abbreviations for the sandy lands and loess samples are as Figs 1 and 2.

The (dis)similarities between the sandy lands can be visually plotted in the MDS diagram in which all heavy mineral types in each sandy lands are taken into consideration. As seen in Fig 5, the SNSL is separated far from both the ODSL and HQSL, but slightly overlaps with the HLSL. The Harbin loess samples are ploted dispersedly, with one sample falling in ODSL field, and the Chifeng, Kulungou and Xingshan loess samples cluster tightly into HQSL field but far from the Harbin loess samples. This observation indicates that an exact source-to-sink relation is not well recognized in the MDS plot presumably due to the fact that some minerals are, to a different extent, diluted or enriched in the loess samples, which can produce a certain noise for data analysis.

It has long been believed that heavy minerals are transferred nearly quantitatively into sedimentary record from source to sink during sedimentary processes and thus would powerfully distinguish source rocks and sediment provenance [63, 64]. In the source-to-sink system in the NESL, however, a marked difference in heavy mineral species between the loess and source deposits can be recognized (Fig 4). Some factors, such as sedimentary differentiation, post-deposition alteration and similar source material composition, mask the source-to-sink correlation and compromise heavy-mineral provenance interpretations. Accordingly, the heavy mineral composition should be used with caution in detecting dust source areas of the NE loess. However, that is possible when considering some geographical elements (e.g., prevailing near-surface wind direction and the source-to-sink spatial position relation, etc). The Harbin loess, for example, is marked by clearly high contents of hematite+limonite and low contents of garnet, which is clearly different from the other NE loess as well as ODSL and HQSL. This observation excludes the ODSL and HQSL as the main dust source of the Harbin loess. However, we cannot definitely identify by the heavy mineral data that whether the SNSL or the HLSL is the dust source of the Harbin loess. Considering the annual dominant wind direction (i.e., southwesterly) in the Songnen Plain [19, 45, 65, 66], we reasonably argue that the SNSL rather than the HLSL fed main dust particles to the Harbin loess, consistent with Sr-Nd isotopic results-deduced recognition [19].

### The influence of river processes on the composition of heavy minerals

Heavy mineral composition in detrital sediments is largely influenced by provenance and transport-deposition processes [67, 68]. It is a common method for provenance tracing that only a few samples (commonly 1), due to the difficulty of sampling, are used to depict the heavy-mineral characteristics of the parent rocks in the entire drainage system [11, 26]. This, however, can lead to biased results.

For the heavy mineral composition of the Balan River (Fig 3 and S4 Table), the eight samples show the significantly diverse heavy mineral composition downstream, with variation coefficients ranging from 0.2 to 1.4 (0.7) for <63 μm, from 0.2 to 2.8 (0.9) for 63–125 μm and from 0.1 to 2.8 (1.1) for 125–250 μm. For 63–125 μm fractions as an example, the ultra-stable heavy minerals, such as zircon, rutile, tourmaline and anatase, show a high coefficient of variation (0.62, 1.62, 1.75, and 0.91, respectively), and both rutile and tourmaline appear only in a few samples, while siderite appears only in one sample. Other heavy minerals also have a high variation coefficient, but sphene and amphibole have a relatively low variation coefficient (0.2 and 0.22, respectively). Due to the distinct differences in the heavy mineral contents, the heavy mineral assemblage of each sample is also varying, shifting among hornblende, ilmenite, magnetite, sphene and epidote. Besides, it is believed that, during sedimentary processes, the relative content of stable heavy minerals increases with increasing transport distance, while the unstable minerals show an opposite trend [31, 69, 70]. However, this trend is not recognized in the Balan River, and the heavy mineral abundances do not show regular changes downstream. Notably, tourmaline, rutile and monazite stably appear only in the downstream.

Despite the same provenance, the downstream sediments in the Balan river show significantly different heavy mineral composition (see Fig 3 and S4 Table), indicating that the river transport-deposition processes have significantly affected the distribution and enrichment of heavy minerals in the sediments downstream. Therefore, it is difficult for one or a few samples to picture the heavy mineral composition of the parent rocks in the source, especially if outcrops of some rock types are only minor distributed in the study area, because an effect of these rock types can be missed when only one or a few samples are available. Accordingly, to obtain more accurate source information, larger numbers of samples are needed to more accurately obtain the average heavy mineral composition of the source rocks in the drainage basin.

In the process of sediment transportation and deposition, coarse and fine particles in sediments will be separated due to their different size, density and shape, resulting in the separation and enrichment of heavy minerals into different grain-size fraction components [63]. For the Balan River, the heavy mineral percentage in the sediments is the highest in 63–125 μm fraction (1.9%-15.8%, 5.8%), followed by 125–250 μm (0.7%-4.7%, 2.1%) and <63 μm fractions (0.6%-4.1%, 1.9%) (S5 Table), but stable heavy minerals (e.g., ZTR index) are the highest in <63 μm fractions, followed by 63–125 μm and 125–250 μm fractions (S6 Table). Specifically, zircon, apatite, rutile, anatase, monazite, pyroxene, amphibole and tourmaline are enriched in the fine-grained fraction (<63 μm) (see S4 Table), with the lower content or a lack of some heavy mineral types in the coarser size fractions. For example, monazite is frequent only in the (<63 μm) fraction; tourmaline is seriously absent in the coarser particles. Sphene, hematite-limonite, magnetite and garnet are more frequent in the coarser fraction (63–125 μm and/or 125–250 μm). There is no obvious enrichment trend for epidote.

**Fig 6:**
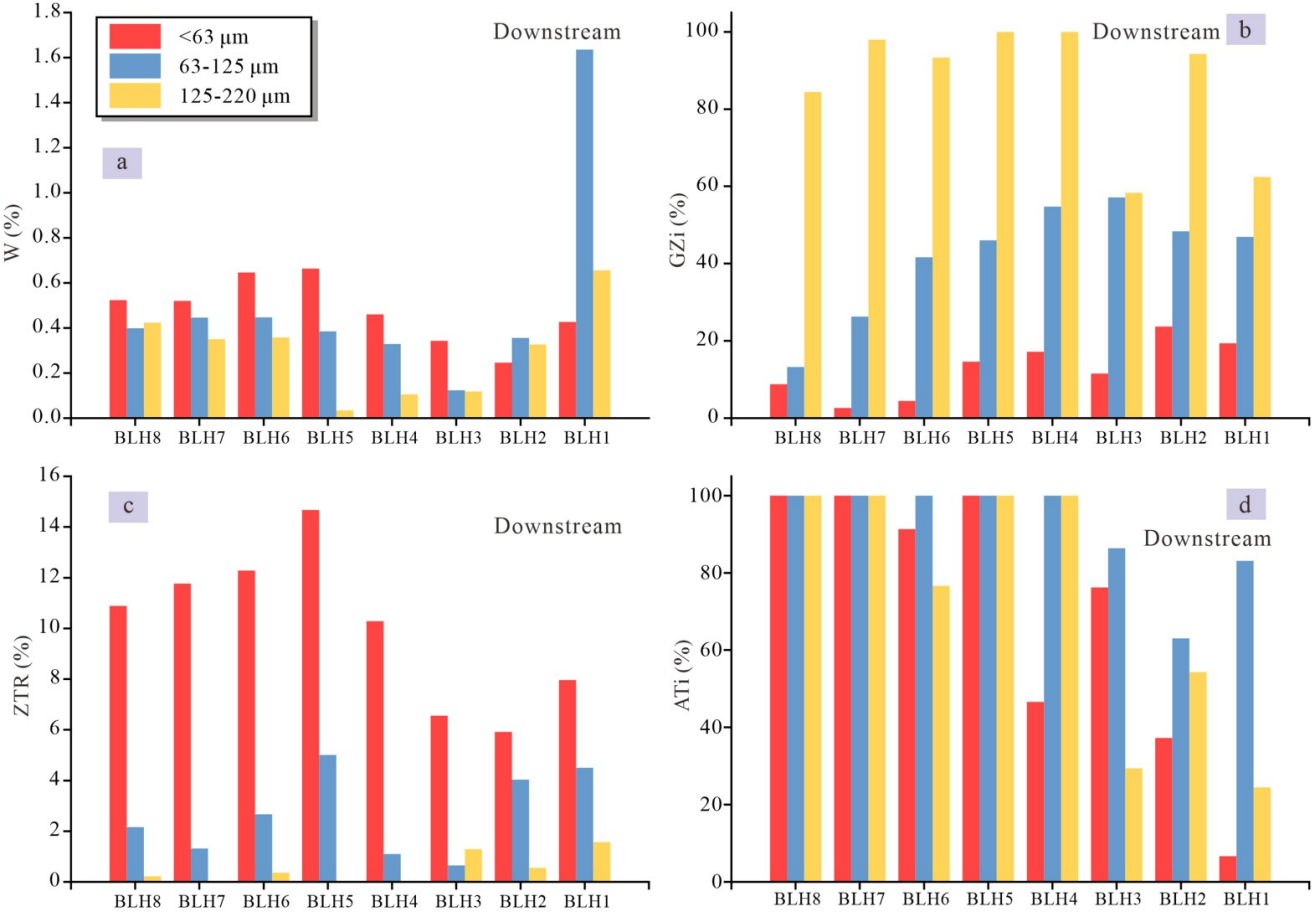
Downstream variations of heavy mineral indexes in the different-sized fractions in the Balan River sediments. (a) W index (%) is the stability coefficient of heavy minerals, W (%) = stable mineral / unstable mineral; (b) GZi (%) = 100 × garnet count / (total garnet plus zircon); (c) ZTR (%) = zircon count + tourmaline count + rutile count; (d) ATi (%) = 100 × apatite count / (total apatite plus tourmaline). Note that the heavy mineral indexes are markedly grain-size dependent, and see text for details.

Ratios of heavy minerals with similar hydraulic and diagenetic behaviour, which are called as heavy mineral characteristic indexes, such as ATi, GZi, RuZi, and MZi, are routinely used to trace the source because they are rarely affected by particle size separation [20, 63]. However, these indexes in this study were significantly affected by particle size. The stability coefficient (W) of heavy minerals (see S7 Table for details about the stability of heavy minerals) in the Balan River drainage shows a decreasing trend overall with an increasing grain size, with the lowest in the 125–250 μm fraction (Fig 6a). The ZTR index is clearly different in the different grain-size fractions, with that in the <63 μm fraction markedly higher than that in the other two fractions due to preferential enrichment of the stable heavy minerals in the smaller grain-size fractions (Fig 6c). The GZi index of the three particle size fractions are different, with a higher value in the coarse-grained fractions due to enrichment of garnet in the 125–250 μm fraction (Fig 6b). The fraction 63–125 μm shows a higher ATi index, while tourmaline is substantially absent in the fraction 63–125 μm and/or 125–250 μm, resulting in no clear variation trend in ATi index (Fig 6d). Therefore, the heavy mineral indexes cannot eliminate or minimize the deviation brought by the grain size effect, an integrated consideration of the different-sized fractions is crucial when interpreting sediment sources.

Very fine sand fraction (63–125 μm) is typically used for heavy mineral-based provenance studies in the river basin [71–73]. The reason for this is that the size fraction of very fine sand (63–125 μm) is rich in heavy minerals [71] and is considered to can be transported by the river for a long distance in long-term suspension [67, 74]. In addition, the fine sand fraction (125–250 μm) is also employed [75, 76], either alone or together with 63–125 μm fraction, for heavy mineral analysis. In this study, the heavy mineral abundances in the sediments are dominantly concentrated in the 63–125 μm fraction, consistent with other studies. However, most of the heavy mineral types (especially stable ones) are mainly enriched in the <63 μm fraction, and the abundances of some heavy mineral types are extremely low and even absent in the coarse-grained fractions (63–125 μm and/or 125–250 μm) (S4 Table). It is thus considered that the hydrodynamic fractionation-induced grain separation, during river transport-deposition processes, has a significant impact on the heavy mineral composition in sediments, and that the heavy mineral characteristics from an individual, specific grain-size range (i.e., a narrow grain-size window) may have lost some important information about the source area, especially if some rock types only produce a specific grain size, which do not occur in the analyzed grain-size window. Therefore, the heavy mineral information retrieved from a narrow grain-size window is difficult to represent the overall characteristics of the parent rocks of the entire basin. A wide grain-size window should be selected when interpreting provenance data to avoid missing the source rock information. The <63 μm and 63–125 μm fractions are suggested, while the 125–250 μm fraction, as a narrow grain-size window, is not suitable for provenance interpretation because some important information may get lost for this fraction.

Therefore, the two aspects should be taken into consideration when interpreting heavy mineral information. First, heavy mineral composition in sediments is markedly dependent on grain size, and thus a narrow grain-size window is not expected to depict the overall characteristics of the drainage basin. A wide grain-size window is suggested for provenance interpretation to avoid the loss of some heavy mineral types hosted in a specific grain-size fraction. Second, the different river reaches with the same clastic source bear distinct characteristics in the heavy mineral composition, indicating that only one or a few samples can not be expected to portray the average composition of the whole river basin. The sample amounts for the heavy mineral analysis must be taken into consideration, and more amounts of samples are needed to obtain more accurate source-rock information.

## Conclusion

In this study, the fine-grained aeolian-fluvial sediments and loess deposits were taken from the NESL to construct the heavy mineral composition of the NESL and evaluate the application of heavy minerals in source-to-sink system. Additionally, the influence of river processes on heavy mineral composition was discussed with the Balan river as an example. The main conclusions are as follows.

1. A moderate similarity in heavy mineral types can be observed in the NESL, especially between the ODSL and HQSL as well as between the HLSL and the SNSL, collectively with ilmenite, epidote, zircon and amphibole as primary minerals. Despite this, some differences are also clear in some characteristic minerals. For example, hematite+limonite and pyroxene discriminate the ODSL and HQSL from the HLSL and SNSL; the ODSL is discriminated from the HQSL by magnetite; the HLSL is distinguished from the SNSL by garnet and to a certain extent amphibole.
2. In the source-to-sink system in the NESL, the heavy mineral composition of the loess deposits and that of the sandy-land sources does not correlate entirely, with different degrees of enrichment or loss, and only a few heavy mineral types are consistent. In this regard, the determination of the provenance of loess by the heavy mineral method is weak unless physical and geographical conditions are taken into account.
3. Even under the same provenance conditions, the sediments in the different Balan River reaches show significantly different heavy mineral composition, indicating that heavy mineral composition in the sediments downstream has been influenced substantially by river transport-deposition processes, which indicates a clear diversity of heavy minerals in different river reaches.
4. The significant heavy-mineral differences can be observed in the different particle size fractions. Zircon, apatite, rutile, anatase, monazite, pyroxene, amphibole and tourmaline are more frequent in the fine particle fraction (<63 μm), while sphene, hematite+limonite, magnetite and garnet are enriched in the coarser particle fractions (63–125 μm and/or 125–250 μm), and there is no significant enrichment pattern for epidote. The heavy mineral indexes are also grain-size-dependent to a great extent.
5. The heavy-mineral weight percentage in the sediments is considerably concentrated in the 63–125 μm fraction, most of the heavy mineral types (especially stable ones) are more frequent in the <63 μm fraction, and the abundances of some heavy mineral types are extremely low and even absent in the coarser fractions (63–125 μm and/or 125–250 μm).
6. In order to obtain a complete picture about the geological framework in the source area, not only the grain-size window, but also the sample amount is of importance for interpreting heavy-mineral data. A wide grain-size window and more amounts of samples are needed.

## Supporting information

S1 Table Description of sampling points. (DOCX)

S2 Table Heavy mineral abundances (wt.%) of the sandy lands in Northeast China. (DOCX)

S3 Table Heavy mineral abundances (wt.%) of the loess deposits in Northeast China. (DOCX)

S4 Table Heavy mineral abundances (wt.%) in the different-sized fractions in the Balan River. (DOCX)

S5 Table Heavy mineral percentage (wt.%) of the different-sized fractions in the Balan River Basin. (DOCX)

S6 Table Variations of heavy mineral indexes of the different-sized fractions in the Balan River Basin. (DOCX)

S7 Table The stability of the more common heavy mineral species. (DOCX)
