# Supplementary material for: The composition of heavy minerals of the sandy lands, Northeast China and their implications for tracing detrital sources
Source: PLoS One. 2022 Oct 20;17(10):e0276494. doi: 10.1371/journal.pone.0276494 (PMC9584371; doi:10.1371/journal.pone.0276494)
Supplement: S1 Table — (DOCX) [file pone.0276494.s001.docx]

**S1 Table. Description of sampling points.**

| Samples | Types | Longitude(E) | Latitude(N) |
| --- | --- | --- | --- |
| OD1 | Fluvial sand | 117°32.202′ | 43°13.180′ |
| OD2 | Fluvial sand | 117°32.787′ | 43°02.972′ |
| OD3 | Fluvial sand | 117°32.276′ | 43°03.708′ |
| OD4 | Fluvial sand | 117°32.362′ | 43°04.690′ |
| OD7 | Fluvial sand | 117°33.305 | 43°09.592′ |
| OD8 | Fluvial sand | 117°33.459′ | 43°09.712′ |
| OD9 | Fluvial sand | 117°31.987′ | 43°13.632′ |
| OD10 | Eolian sands | 117°16.802′ | 43°10.579′ |
| OD12 | Eolian sands | 117°00.914′ | 43°03.086′ |
| OD14 | Eolian sands | 115°50.451′ | 42°46.313′ |
| OD17 | Eolian sands | 114°49.796′ | 42°32.394′ |
| OD23 | Fluvial sand | 117°00.914′ | 43°03.086′ |
| OD25 | Eolian sands | 117°18′19.1″ | 43°12′05.2″ |
| HQ3 | Fluvial sediments | 121°00.561′ | 43°36.634′ |
| HQ6 | Fluvial sediments | 120°48.457′ | 43°25.527′ |
| HQ9 | Fluvial sediments | 120°28.930′ | 43°09.428′ |
| HQ10 | Fluvial sediments | 120°33.310′ | 43°12.361′ |
| HQ11 | Eolian sands | 120°36.968′ | 43°10.254′ |
| HQ12 | Eolian sands | 120°46.336′ | 43°03.853′ |
| HQ13 | Eolian sands | 120°42.245′ | 42°53.921′ |
| HQ14 | Eolian sands | 121°10.250′ | 43°06.403′ |
| HQ22 | Eolian sands | 121°59.729′ | 42°51.544′ |
| SN62 | Fluvial sediments | 123°10.107′ | 47°05.255′ |
| SN73 | Fluvial sediments | 123°32.338′ | 47°06.370′ |
| Ha22 | Fluvial sediments | 126°10.585′ | 45°08.791′ |
| JL44 | Fluvial sediments | 126°01.047′ | 44°47.959′ |
| SN40a | Fluvial sediments | 124°51.478′ | 45°40.082′ |
| HL30 | Eolian sands | 119°42.800′ | 49°01.016 |
| HL33 | Eolian sands | 119°48.261′ | 48°57.498′ |
| HL34 | Eolian sands | 119°04.132′ | 47°47.504′ |
| HL35 | Eolian sands | 119°00.070′ | 47°49.558′ |
| HL36 | Fluvial sediments | 118°21.586′ | 48°14.092′ |
| HL37 | Fluvial sediments | 118°35.679′ | 48°23.212′ |
| Chifeng (CF) | Loess | 118.70 | 42.31 |
| Kulungou (KLG) | Loess | 121.78 | 42.73 |
| Harbin (HB) | Loess | 126.80 | 45.80 |
| Xingshan (XS) | Loess | 124.36 | 43.26 |
| Balanhe (BLH) | Fluvial sediments | * | * |
