# Supplementary material for: The composition of heavy minerals of the sandy lands, Northeast China and their implications for tracing detrital sources
Source: PLoS One. 2022 Oct 20;17(10):e0276494. doi: 10.1371/journal.pone.0276494 (PMC9584371; doi:10.1371/journal.pone.0276494)
Supplement: S2 Table — (DOCX) [file pone.0276494.s002.docx]

**S2 Table. Heavy mineral abundances (wt.%) of the sandy lands in Northeast China.**

| Sample | Zr | Ap | Rt | Spn | Leu | Ky | Ant | Gt | Mnz | Tur | Ep | Px | Amp | Ilm | Tmt | Hem+Lm | Mag | Others |
| --- | --- | --- | --- | --- | --- | --- | --- | --- | --- | --- | --- | --- | --- | --- | --- | --- | --- | --- |
| OD1 | 4.75 | 0.00 | 0.38 | 0.29 | 0.45 | 0.00 | 0.35 | 6.89 | 0.22 | 2.67 | 15.1 | 11.7 | 9.89 | 22.6 | 4.71 | 3.11 | 9.61 | 7.34 |
| OD2 | 17.2 | 1.87 | 1.65 | 2.20 | 0.88 | 0.00 | 2.09 | 5.21 | 1.54 | 3.75 | 11.2 | 9.99 | 5.29 | 17.0 | 5.13 | 3.45 | 4.62 | 6.81 |
| OD3 | 4.76 | 3.80 | 2.23 | 1.32 | 1.89 | 0.00 | 1.00 | 13.2 | 0.00 | 1.80 | 17.6 | 0.00 | 8.52 | 30.7 | 0.00 | 1.55 | 0.00 | 11.7 |
| OD4 | 15.4 | 3.11 | 1.26 | 0.68 | 1.36 | 0.00 | 0.97 | 2.23 | 1.65 | 1.84 | 11.5 | 13.3 | 12.8 | 22.4 | 2.10 | 2.91 | 2.47 | 3.97 |
| OD7 | 7.92 | 0.53 | 0.91 | 0.56 | 0.47 | 0.00 | 0.82 | 3.53 | 0.35 | 1.95 | 7.55 | 16.6 | 14.7 | 18.1 | 8.93 | 8.28 | 5.81 | 2.97 |
| OD8 | 7.73 | 0.80 | 0.72 | 0.30 | 0.63 | 0.00 | 0.58 | 6.65 | 0.36 | 2.70 | 9.36 | 6.93 | 9.15 | 16.7 | 16.6 | 5.20 | 12.6 | 3.08 |
| OD9 | 18.0 | 1.89 | 0.91 | 0.66 | 1.32 | 0.00 | 1.48 | 4.08 | 0.99 | 4.26 | 8.95 | 6.78 | 6.00 | 26.3 | 3.27 | 2.43 | 9.53 | 3.17 |
| OD10 | 6.03 | 0.41 | 0.58 | 0.15 | 0.46 | 0.00 | 0.24 | 7.13 | 0.29 | 6.92 | 9.54 | 12.3 | 8.91 | 19.1 | 16.0 | 2.20 | 5.71 | 4.08 |
| OD12 | 14.5 | 1.30 | 1.01 | 1.16 | 0.94 | 0.00 | 1.23 | 7.35 | 0.79 | 2.89 | 7.45 | 6.23 | 5.95 | 14.2 | 17.7 | 1.67 | 11.1 | 4.52 |
| OD14 | 9.27 | 0.38 | 1.03 | 0.80 | 0.69 | 0.00 | 1.22 | 8.43 | 0.23 | 3.85 | 9.36 | 6.76 | 8.22 | 27.4 | 6.96 | 4.89 | 5.66 | 4.91 |
| OD17 | 3.03 | 0.16 | 0.21 | 0.27 | 0.12 | 0.00 | 0.32 | 5.46 | 0.25 | 4.77 | 9.61 | 10.5 | 9.31 | 18.5 | 19.2 | 3.61 | 10.1 | 4.55 |
| OD23 | 7.94 | 2.24 | 1.28 | 1.09 | 1.86 | 0.00 | 1.86 | 4.22 | 0.96 | 3.37 | 13.4 | 8.33 | 10.3 | 20.1 | 9.28 | 3.48 | 4.55 | 5.68 |
| OD25 | 9.84 | 1.02 | 1.19 | 0.76 | 0.89 | 0.00 | 1.48 | 8.41 | 0.72 | 3.03 | 12.6 | 10.8 | 4.11 | 20.8 | 10.4 | 4.60 | 4.02 | 5.33 |
| HQ3 | 4.68 | 1.00 | 2.22 | 1.46 | 0.63 | 0.00 | 1.34 | 17.6 | 0.58 | 0.88 | 22.82 | 8.78 | 7.90 | 24.6 | 0 | 5.27 | 0.00 | 0.31 |
| HQ6 | 5.52 | 1.31 | 2.10 | 1.71 | 0.66 | 0.00 | 0.92 | 10.4 | 0.26 | 3.47 | 17.4 | 11.3 | 8.69 | 21.7 | 0 | 7.82 | 0.00 | 6.74 |
| HQ9 | 7.58 | 0.16 | 1.32 | 2.31 | 0.82 | 0.00 | 1.81 | 10.0 | 1.15 | 0.84 | 12.5 | 10.9 | 9.19 | 24.2 | 0 | 10.0 | 0.00 | 7.16 |
| HQ10 | 7.54 | 6.54 | 3.77 | 3.52 | 0.50 | 0.00 | 1.76 | 19.5 | 1.26 | 1.50 | 18.0 | 5.99 | 0.75 | 22.5 | 0 | 5.24 | 0.00 | 1.75 |
| HQ11 | 9.09 | 1.59 | 4.32 | 0.00 | 1.14 | 0.00 | 1.36 | 17.0 | 0.00 | 0.77 | 20.1 | 5.41 | 3.86 | 23.2 | 0 | 3.09 | 0.00 | 9.09 |
| HQ12 | 10.9 | 3.62 | 3.14 | 2.17 | 0.03 | 0.00 | 1.69 | 12.2 | 0.97 | 0.82 | 10.6 | 13.7 | 8.97 | 22.0 | 0 | 3.06 | 0.00 | 6.14 |
| HQ13 | 2.83 | 1.62 | 1.21 | 0.91 | 0.40 | 0.00 | 1.31 | 8.09 | 0.71 | 4.49 | 34.2 | 14.4 | 11.7 | 8.99 | 0 | 2.70 | 0.00 | 6.51 |
| HQ14 | 6.26 | 1.19 | 2.09 | 2.68 | 0.30 | 0.00 | 0.89 | 10.2 | 0.60 | 0.85 | 11.9 | 5.96 | 11.1 | 32.3 | 0 | 8.51 | 0.00 | 5.15 |
| HQ22 | 5.23 | 0.29 | 1.14 | 0.95 | 0.90 | 0.00 | 0.86 | 14.5 | 0.38 | 2.71 | 16.3 | 10.9 | 8.14 | 27.2 | 0 | 4.52 | 0.00 | 6.10 |

**Table S2 Continued.**

| Sample | Zr | Ap | Rt | Spn | Leu | Ky | Ant | Gt | Mnz | Tur | Ep | Px | Amp | Ilm | Tmt | Hem+Lm | Mag | Others |
| --- | --- | --- | --- | --- | --- | --- | --- | --- | --- | --- | --- | --- | --- | --- | --- | --- | --- | --- |
| SN62 | 7.68 | 0.37 | 0.61 | 2.86 | 1.27 | 0.00 | 0.25 | 3.60 | 0.37 | 0.54 | 37.8 | 0.00 | 7.19 | 17.1 | 0 | 12.6 | 2.74 | 5.12 |
| SN73 | 4.74 | 1.26 | 0.54 | 4.56 | 0.61 | 0.00 | 0.11 | 1.28 | 0.30 | 0.14 | 19.6 | 0.00 | 3.70 | 11.5 | 0 | 22.1 | 24.3 | 5.25 |
| Ha22 | 11.01 | 0.97 | 0.48 | 2.20 | 0.45 | 0.00 | 0.27 | 0.62 | 0.00 | 0.00 | 13.5 | 2.49 | 44.0 | 6.65 | 0 | 4.36 | 8.03 | 4.95 |
| JL44 | 1.99 | 2.20 | 0.44 | 3.13 | 0.82 | 0.00 | 0.25 | 0.59 | 0.00 | 1.17 | 16.8 | 0.82 | 13.1 | 6.33 | 0 | 29.3 | 18.4 | 4.53 |
| SN40a | 7.92 | 2.00 | 1.31 | 5.86 | 0.87 | 0.00 | 0.06 | 0.52 | 0.00 | 0.00 | 28.2 | 0.00 | 21.4 | 16.8 | 0 | 4.80 | 9.69 | 5.38 |
| HL30 | 10.3 | 2.44 | 0.29 | 0.19 | 2.36 | 0.00 | 0.91 | 0.30 | 0.91 | 0.30 | 8.34 | 0.00 | 0.91 | 11.7 | 0 | 26.5 | 27.7 | 6.89 |
| HL33 | 6.24 | 1.73 | 1.16 | 1.44 | 1.78 | 0.25 | 0.33 | 4.25 | 0.21 | 1.31 | 23.4 | 0.00 | 19.5 | 21.3 | 0 | 5.72 | 2.19 | 9.31 |
| HL34 | 7.63 | 0.74 | 1.23 | 2.74 | 0.40 | 0.00 | 0.03 | 21.6 | 0.26 | 0.99 | 9.66 | 0.99 | 10.9 | 25.8 | 0 | 6.94 | 0.00 | 10.1 |
| HL35 | 11.8 | 0.79 | 2.00 | 1.42 | 1.07 | 0.00 | 0.23 | 12.9 | 0.28 | 1.04 | 12.9 | 0.35 | 6.10 | 27.9 | 0 | 5.58 | 7.20 | 8.47 |
| HL36 | 9.33 | 0.51 | 0.91 | 3.13 | 1.93 | 0.12 | 0.12 | 11.3 | 0.24 | 2.62 | 14.7 | 0.70 | 10.3 | 27.6 | 0 | 5.41 | 2.07 | 9.07 |
| HL37 | 8.02 | 0.71 | 0.93 | 1.64 | 1.36 | 0.06 | 0.22 | 9.42 | 0.09 | 0.91 | 10.5 | 0.54 | 8.70 | 14.1 | 0 | 31.7 | 3.89 | 7.17 |

.

Systematic mineral abbreviation list: Ilm=ilmenite, Ep=epidote, Gt=garnet, Px=pyroxene, Mag=Magnetite, Amp=amphibole, Zr=zircon, Hem=hematite, Lm=limonite, Spn=sphene, Tur=tourmaline, Tmt=titanomagnetite, Ap=apatite, Rt=rutile, Leu=leucoxene, Ant=anatase, Mnz=monazite, Ky=kyanite, other=weathered debris. Some heavy minerals, such as giauconie, pyrite, kyanite and xenotime, only present sporadically with extremely low amounts (1-13 grains) in one or a few samples, and thus are not listed in the table.
