# Supplementary material for: The composition of heavy minerals of the sandy lands, Northeast China and their implications for tracing detrital sources
Source: PLoS One. 2022 Oct 20;17(10):e0276494. doi: 10.1371/journal.pone.0276494 (PMC9584371; doi:10.1371/journal.pone.0276494)
Supplement: S3 Table — (DOCX) [file pone.0276494.s003.docx]

**S3 Table. Heavy mineral abundances (wt.%) of the loess deposits in Northeast China.**

| Sample | Zr | Ap | Rt | Spn | Leu | Ky | Gt | Ant | Mnz | Tur | Ep | Px | Amp | Ilm | Tmt | Hem+Lm | Mag | Others |
| --- | --- | --- | --- | --- | --- | --- | --- | --- | --- | --- | --- | --- | --- | --- | --- | --- | --- | --- |
| CF10 | 11.2 | 0.15 | 1.27 | 5.93 | 0.51 | 0.04 | 15.3 | 0.07 | 0.15 | 0.29 | 18.9 | 1.16 | 7.57 | 19.37 | 0 | 2.91 | 11.8 | 3.35 |
| CF11 | 8.23 | 0.36 | 0.94 | 5.75 | 0.87 | 0.03 | 13.4 | 0.13 | 0.15 | 0.15 | 15.2 | 3.53 | 7.82 | 24.85 | 0 | 2.30 | 11.7 | 4.64 |
| CF12 | 7.56 | 0.12 | 1.15 | 5.95 | 0.76 | 0.06 | 18.4 | 0.06 | 0.16 | 0.47 | 16.3 | 1.40 | 10.42 | 21.92 | 0 | 1.55 | 9.19 | 4.43 |
| CF13 | 7.48 | 0.13 | 1.93 | 4.24 | 1.34 | 0.03 | 18.1 | 0.10 | 0.48 | 0.64 | 23.2 | 2.56 | 5.59 | 20.61 | 0 | 0.96 | 8.67 | 3.98 |
| KLG10 | 8.67 | 0.37 | 1.53 | 12.4 | 0.53 | 0.03 | 17.7 | 0.09 | 1.41 | 0.70 | 17.7 | 2.81 | 7.03 | 21.78 | 0 | 1.93 | 2.04 | 3.18 |
| KLG11 | 8.45 | 0.43 | 1.50 | 8.15 | 0.63 | 0.03 | 14.5 | 0.14 | 0.82 | 0.33 | 22.3 | 1.79 | 4.89 | 24.63 | 0 | 1.14 | 6.64 | 3.58 |
| KLG12 | 7.85 | 0.41 | 1.83 | 6.93 | 0.41 | 0.03 | 13.6 | 0.03 | 0.17 | 0.50 | 22.5 | 3.03 | 4.04 | 27.75 | 0 | 2.52 | 4.47 | 3.84 |
| KLG13 | 4.83 | 0.35 | 1.46 | 6.35 | 0.96 | 0.02 | 16.7 | 0.10 | 0.72 | 0.90 | 31.4 | 2.33 | 5.92 | 20.62 | 0 | 0.72 | 4.20 | 2.47 |
| HB2 | 1.92 | 0.00 | 0.15 | 0.74 | 0.27 | 0.00 | 2.37 | 0.12 | 0.00 | 0.22 | 13.8 | 0.00 | 9.26 | 18.47 | 0 | 28.9 | 15.5 | 7.72 |
| HB4 | 9.85 | 0.56 | 0.89 | 2.02 | 0.92 | 0.00 | 6.98 | 0.36 | 0.03 | 0.31 | 12.5 | 0.31 | 4.73 | 5.50 | 0 | 39.3 | 10.1 | 5.63 |
| HB5 | 10.0 | 0.47 | 0.71 | 1.28 | 0.67 | 0.00 | 3.84 | 0.37 | 0.03 | 0.14 | 10.6 | 1.37 | 1.92 | 4.67 | 0 | 49.1 | 9.17 | 5.63 |
| HB7 | 7.81 | 0.57 | 0.54 | 6.03 | 1.31 | 0.00 | 2.72 | 0.17 | 0.00 | 0.00 | 15.6 | 0.00 | 18.34 | 30.40 | 0 | 7.13 | 5.27 | 4.31 |
| XS1 | 13.1 | 0.04 | 1.20 | 1.96 | 0.44 | 0.00 | 8.48 | 0.41 | 0.17 | 1.90 | 25.3 | 0.52 | 0.17 | 38.44 | 0 | 3.81 | 1.17 | 2.91 |

Systematic mineral abbreviation list: Ilm=ilmenite, Ep=epidote, Gt=garnet, Px=pyroxene, Mag=Magnetite, Amp=amphibole, Zr=zircon, Hem=hematite, Lm=limonite, Spn=sphene, Tur=tourmaline, Ap=apatite, Rt=rutile, Leu=leucoxene, Ant=anatase, Mnz=monazite, Ky=kyanite, other=weathered debris. Some heavy minerals, such as pyrite, chalcopyrite and moissanite, only present sporadically with extremely low amounts (1-40 grains) in one or a few samples, and thus are not listed in the table.
