# Supplementary material for: The composition of heavy minerals of the sandy lands, Northeast China and their implications for tracing detrital sources
Source: PLoS One. 2022 Oct 20;17(10):e0276494. doi: 10.1371/journal.pone.0276494 (PMC9584371; doi:10.1371/journal.pone.0276494)
Supplement: S4 Table — (DOCX) [file pone.0276494.s004.docx]

**S4 Table. Heavy mineral abundances (wt.%) in the different-sized fractions in the Balan River.**

| Sample | Zr | Ap | Rt | Ant | Mnz | Px | Amp | Tur |  | HemLm | Mag | Mgh | Gt |  | Spn | Ep | Leu |
| --- | --- | --- | --- | --- | --- | --- | --- | --- | --- | --- | --- | --- | --- | --- | --- | --- | --- |
| BLH1 A | 7.00 | 0.06 | 0.12 | 0.06 | 0.17 | 2.85 | 50.70 | 0.84 |  | 3.02 | 4.1 | 0 | 1.68 |  | 12.41 | 1.18 | 0.42 |
| BLH2 A | 4.76 | 0.63 | 0.10 | 0.03 | 0.21 | 4.86 | 55.97 | 1.06 |  | 0.42 | 4.29 | 0.48 | 1.48 |  | 9.42 | 3.17 | 0.02 |
| BLH3 A | 6.05 | 1.25 | 0.12 | 0.09 | 0.20 | 2.95 | 48.94 | 0.39 |  | 1.38 | 1.57 | 2.48 | 0.79 |  | 15.81 | 4.13 | 0.20 |
| BLH4 A | 9.14 | 0.83 | 0.19 | 0.69 | 0.00 | 2.47 | 38.17 | 0.95 |  | 3.60 | 3.05 | 2.07 | 1.90 |  | 9.80 | 15.00 | 0.19 |
| BLH5 A | 14.11 | 1.00 | 0.56 | 0.83 | 0.00 | 0.00 | 30.75 | 0.00 |  | 1.61 | 0.56 | 10.42 | 2.42 |  | 9.14 | 14.28 | 0.72 |
| BLH6 A | 11.93 | 2.00 | 0.16 | 0.05 | 0.00 | 0.56 | 42.51 | 0.19 |  | 1.50 | 9.18 | 2.51 | 0.56 |  | 13.52 | 3.56 | 0.05 |
| BLH7 A | 11.53 | 2.10 | 0.24 | 0.05 | 0.00 | 1.85 | 44.78 | 0.00 |  | 1.39 | 12.31 | 3.13 | 0.31 |  | 8.23 | 7.10 | 0.20 |
| BLH8 A | 10.72 | 1.37 | 0.17 | 0.05 | 0.00 | 0.41 | 44.02 | 0.00 |  | 0.62 | 3.41 | 5.23 | 1.03 |  | 14.37 | 5.14 | 0.11 |
| Average | 9.41 | 1.16 | 0.21 | 0.23 | 0.07 | 1.99 | 44.48 | 0.43 |  | 1.69 | 4.80 | 3.29 | 1.27 |  | 11.59 | 6.70 | 0.24 |
| BLH1B | 4.29 | 0.64 | 0.08 | 0.06 | 0.00 | 0.92 | 22.12 | 0.13 |  | 3.40 | 15.5 | 6.66 | 3.79 |  | 16.35 | 4.19 | 0.17 |
| BLH2 B | 3.45 | 0.92 | 0.04 | 0.07 | 0.00 | 1.98 | 43.67 | 0.54 |  | 1.80 | 3.33 | 9.07 | 3.23 |  | 10.09 | 7.19 | 0.18 |
| BLH3 B | 0.45 | 1.27 | 0.00 | 0.01 | 0.00 | 1.00 | 55.06 | 0.20 |  | 2.80 | 0.27 | 5.9 | 0.60 |  | 18.37 | 6.61 | 0.01 |
| BLH4 B | 1.10 | 0.25 | 0.00 | 0.11 | 0.00 | 1.56 | 46.22 | 0.00 |  | 1.33 | 9.16 | 2.74 | 1.33 |  | 16.30 | 7.78 | 0.29 |
| BLH5 B | 5.01 | 0.42 | 0.00 | 0.21 | 0.00 | 0.25 | 45.18 | 0.00 |  | 2.76 | 3.97 | 3.58 | 4.27 |  | 14.09 | 11.30 | 0.27 |
| BLH6 B | 2.65 | 0.34 | 0.02 | 0.10 | 0.00 | 0.52 | 40.64 | 0.00 |  | 3.96 | 2.62 | 17.75 | 1.89 |  | 15.50 | 1.72 | 0.34 |
| BLH7 B | 1.32 | 1.78 | 0.00 | 0.02 | 0.00 | 0.78 | 48.36 | 0.00 |  | 1.56 | 12.75 | 8.48 | 0.47 |  | 10.50 | 2.65 | 0.20 |
| BLH8 B | 2.16 | 1.29 | 0.00 | 0.01 | 0.00 | 0.49 | 50.50 | 0.00 |  | 1.80 | 8.21 | 6.39 | 0.33 |  | 14.29 | 2.46 | 0.18 |
| Average | 2.55 | 0.86 | 0.02 | 0.07 | 0.00 | 0.94 | 43.97 | 0.11 |  | 2.43 | 6.97 | 7.57 | 1.99 |  | 14.44 | 5.49 | 0.21 |
| BLH1 C | 1.21 | 0.11 | 0.02 | 0.02 | 0.00 | 0.50 | 36.21 | 0.34 |  | 3.52 | 4.52 | 2.74 | 2.01 |  | 26.99 | 2.68 | 0.09 |
| BLH2 C | 0.16 | 0.44 | 0.02 | 0.02 | 0.00 | 0.75 | 38.78 | 0.37 |  | 1.87 | 1.13 | 2.38 | 2.62 |  | 35.97 | 3.00 | 0.12 |
| BLH3 C | 0.45 | 0.35 | 0.00 | 0.00 | 0.00 | 1.26 | 53.39 | 0.84 |  | 1.05 | 1.28 | 0 | 0.63 |  | 28.77 | 3.99 | 0.14 |
| BLH4 C | 0.00 | 1.39 | 0.00 | 0.00 | 0.00 | 1.46 | 39.49 | 0.00 |  | 2.30 | 1.74 | 1.87 | 1.05 |  | 30.36 | 7.73 | 4.81 |
| BLH5 C | 0.00 | 1.03 | 0.00 | 0.10 | 0.00 | 0.53 | 45.00 | 0.00 |  | 0.27 | 1.08 | 0 | 0.53 |  | 12.04 | 26.21 | 2.12 |
| BLH6 C | 0.18 | 0.59 | 0.00 | 0.12 | 0.00 | 0.18 | 42.67 | 0.18 |  | 2.88 | 0.98 | 4.32 | 2.52 |  | 26.47 | 3.42 | 0.45 |
| BLH7 C | 0.01 | 0.75 | 0.00 | 0.00 | 0.00 | 0.16 | 49.43 | 0.00 |  | 1.43 | 14.39 | 3.11 | 0.48 |  | 18.47 | 4.77 | 0.19 |
| BLH8 C | 0.22 | 0.29 | 0.00 | 0.00 | 0.00 | 0.17 | 42.40 | 0.00 |  | 1.02 | 6.02 | 5.87 | 1.19 |  | 26.45 | 2.71 | 0.22 |
| Average | 0.28 | 0.62 | 0.01 | 0.03 | 0.00 | 0.63 | 43.42 | 0.22 |  | 1.79 | 3.89 | 2.53 | 1.38 |  | 25.69 | 6.81 | 1.02 |
| Enrichment Enriched in fine particles | | | | | | | | |  | Enriched in intermediate particle | | | |  | Enriched in coarse-grained | | |

In the table：A: < 63 μm, B: 63-125 μm, C: 125-250 μm. Systematic mineral abbreviation list: Amp=amphibole, Spn=sphene, Zr=zircon, Mgh=maghemite, Ilm=ilmenite, Mag=Magnetite, Hem=hematite, Lm=limonite, Px=pyroxene, Gt=garnet, Ep=epidote, Tur=tourmaline, Leu=leucoxene, Mnz=monazite, Rt=rutile, Ant=anatase, Ap=apatite, other=weathered debris. Some heavy minerals, such as siderite, pyrite, moissanite, chrome spinel, fluorite and gold, only present sporadically with extremely low amounts (1-12 grains) in one or a few samples, and thus are not listed in the table.
