# Supplementary material for: The composition of heavy minerals of the sandy lands, Northeast China and their implications for tracing detrital sources
Source: PLoS One. 2022 Oct 20;17(10):e0276494. doi: 10.1371/journal.pone.0276494 (PMC9584371; doi:10.1371/journal.pone.0276494)
Supplement: S5 Table — (DOCX) [file pone.0276494.s005.docx]

**S5 Table. Heavy mineral percentage (wt.%) of the different-sized fractions in the Balan River Basin.**

| Grain size (μm) | Sample ID | Original weight (g) | Heavy part content (mg) | Percentage of weight (*100) |
| --- | --- | --- | --- | --- |
| <63 | BLH1 | 39 | 981 | 2.515 |
|  | BLH2 | 31 | 585 | 1.887 |
|  | BLH3 | 28 | 338 | 1.207 |
|  | BLH4 | 8 | 96 | 1.200 |
|  | BLH5 | 6 | 36 | 0.600 |
|  | BLH6 | 12 | 489 | 4.075 |
|  | BLH7 | 28 | 646 | 2.307 |
|  | BLH8 | 24 | 275 | 1.145 |
| 63-125 | BLH1 | 114 | 8834 | 7.749 |
|  | BLH2 | 57 | 2984 | 5.235 |
|  | BLH3 | 46 | 1231 | 2.676 |
|  | BLH4 | 19 | 365 | 1.921 |
|  | BLH5 | 6 | 132 | 2.200 |
|  | BLH6 | 58 | 9174 | 15.81 |
|  | BLH7 | 56 | 3381 | 6.037 |
|  | BLH8 | 35 | 1538 | 4.394 |
| 125-250 | BLH1 | 146 | 6892 | 4.720 |
|  | BLH2 | 176 | 2718 | 1.544 |
|  | BLH3 | 297 | 2031 | 0.683 |
|  | BLH4 | 104 | 907 | 0.872 |
|  | BLH5 | 51 | 411 | 0.805 |
|  | BLH6 | 193 | 7697 | 3.988 |
|  | BLH7 | 158 | 3576 | 2.263 |
|  | BLH8 | 77 | 1526 | 1.981 |
