# Supplementary material for: The composition of heavy minerals of the sandy lands, Northeast China and their implications for tracing detrital sources
Source: PLoS One. 2022 Oct 20;17(10):e0276494. doi: 10.1371/journal.pone.0276494 (PMC9584371; doi:10.1371/journal.pone.0276494)
Supplement: S6 Table — (DOCX) [file pone.0276494.s006.docx]

**S6 Table. Variations of heavy mineral indexes of the different-sized fractions in the Balan River Basin.**

| Grain size (μm) | Sample ID | W | ZTR | Ati | Gzi |
| --- | --- | --- | --- | --- | --- |
| <63 | BLH1 | 0.427 | 7.960 | 6.667 | 19.35 |
|  | BLH2 | 0.247 | 5.920 | 37.27 | 23.71 |
|  | BLH3 | 0.343 | 6.560 | 76.22 | 11.55 |
|  | BLH4 | 0.460 | 10.28 | 46.62 | 17.21 |
|  | BLH5 | 0.663 | 14.67 | 100.0 | 14.64 |
|  | BLH6 | 0.647 | 12.28 | 91.32 | 4.484 |
|  | BLH7 | 0.521 | 11.77 | 100.0 | 2.618 |
|  | BLH8 | 0.524 | 10.89 | 100.0 | 8.766 |
| 63-125 | BLH1 | 1.635 | 4.500 | 83.11 | 46.90 |
|  | BLH2 | 0.355 | 4.030 | 63.01 | 48.35 |
|  | BLH3 | 0.124 | 0.650 | 86.39 | 57.14 |
|  | BLH4 | 0.329 | 1.100 | 100.0 | 54.73 |
|  | BLH5 | 0.385 | 5.010 | 100.0 | 46.01 |
|  | BLH6 | 0.447 | 2.670 | 100.0 | 41.63 |
|  | BLH7 | 0.447 | 1.320 | 100.0 | 26.25 |
|  | BLH8 | 0.399 | 2.160 | 100.0 | 13.25 |
| 125-250 | BLH1 | 0.655 | 1.570 | 24.44 | 62.42 |
|  | BLH2 | 0.327 | 0.550 | 54.32 | 94.24 |
|  | BLH3 | 0.118 | 1.290 | 29.41 | 58.33 |
|  | BLH4 | 0.106 | 0.000 | 100.0 | 100.0 |
|  | BLH5 | 0.034 | 0.000 | 100.0 | 100.0 |
|  | BLH6 | 0.358 | 0.360 | 76.62 | 93.33 |
|  | BLH7 | 0.351 | 0.010 | 100.0 | 97.95 |
|  | BLH8 | 0.424 | 0.220 | 100.0 | 84.39 |

W=stable mineral/unstable mineral; GZi=100×garnet count/(total garnet plus zircon); ZTR=zircon count+tourmaline count+rutile count; ATi=100×apatite count/(total apatite plus tourmaline).
