# Supplementary material for: The composition of heavy minerals of the sandy lands, Northeast China and their implications for tracing detrital sources
Source: PLoS One. 2022 Oct 20;17(10):e0276494. doi: 10.1371/journal.pone.0276494 (PMC9584371; doi:10.1371/journal.pone.0276494)
Supplement: S7 Table — (DOCX) [file pone.0276494.s007.docx]

**S7 Table. The stability of the more common heavy mineral species [1.2].**

| Ultrastable  Stable | rutile, zircon. tourmaline, anatase, apatite  garnet (iron-poor), staurolite, monazite,  biotite, ilmenite, magnetite |
| --- | --- |
| Moderately stable | epidote, kyanite, garnet (iron-rich),  sillimanite, sphene, zoisite |
| Unstable | hornblende, actinolite, pyroxene,  diopside, hypersthene, andalusite |
| Very unstable | olivine |

Reference from (Pettijohn, 1941; Pettijohn et al., 2012).

1.Pettijohn FJ, Potter PE, Siever R. Sand and sandstone. Springer Science & Business Media, 2012.

2.Pettijohn FJ. Persistence of heavy minerals and geologic age. The Journal of Geology. 1941; 49(6): 610-625. https://doi.org/10.1086/624992
